# MiR-21-5p and miR-126a-3p levels in plasma and circulating angiogenic cells: relationship with type 2 diabetes complications

**DOI:** 10.18632/oncotarget.6164

**Published:** 2015-10-19

**Authors:** Fabiola Olivieri, Liana Spazzafumo, Massimiliano Bonafè, Rina Recchioni, Francesco Prattichizzo, Fiorella Marcheselli, Luigina Micolucci, Emanuela Mensà, Angelica Giuliani, Gabriele Santini, Mirko Gobbi, Raffaella Lazzarini, Massimo Boemi, Roberto Testa, Roberto Antonicelli, Antonio Domenico Procopio, Anna Rita Bonfigli

**Affiliations:** ^1^ Department of Clinical and Molecular Sciences, DISCLIMO, Università Politecnica delle Marche, Ancona, Italy; ^2^ Center of Clinical Pathology and Innovative Therapy, INRCA-IRCCS National Institute, Ancona, Italy; ^3^ Center of Biostatistics, INRCA-IRCCS National Institute, Ancona, Italy; ^4^ Department of Experimental, Diagnostic and Specialty Medicine, DIMES, University of Bologna, Bologna, Italy; ^5^ Metabolic Diseases and Diabetology Unit, INRCA-IRCCS National Institute, Ancona, Italy; ^6^ Experimental Models in Clinical Pathology, INRCA-IRCCS National Institute, Ancona, Italy; ^7^ UTIC-Cardiology Unit, INRCA-IRCCS National Institute, Ancona, Italy; ^8^ Scientific Direction, INRCA-IRCCS, National Institute, Ancona, Italy

**Keywords:** miR-21, miR-126, type 2 diabetes, diabetes complication, circulating miRNAs, Gerotarget

## Abstract

Innovative biomarkers are required to manage type 2 diabetic patients (T2DM). We focused our study on miR-126-3p and miR-21-5p levels, as biomarkers of endothelial function and inflammation. MiRNAs levels were measured in plasma from 107 healthy subjects (CTR) and 193 diabetic patients (T2DM), 76 without (T2DM NC) and 117 with (T2DM C) complications.

When diabetic complication were analysed as a whole, miR-126-3p and miR-21-5p levels declined significantly from CTR to T2DM NC and T2DM C patients. When miRNAs levels were related to specific complications, significantly higher miR-21-5p levels (0.46 ± 0.44 vs. 0.26±0.33, *p* < 0.001) and significant lower miR-126-3p levels (0.21±0.21 *vs*. 0.28±0.22, *p* = 0.032) were found in T2DM with previous major cardiovascular events (MACE) *vs.* all the others T2DM patients.

To confirm these results we focused on circulating angiogenic cells (CACs) from a subgroup of 10 CTR, 15 T2DM NC and 15 T2DM patients with MACE. CACs from T2DM patients expressed higher miR-21-5p and lower miR-126-3p levels than CACs from CTR. Furthermore, CACs from T2DM + MACE showed the highest levels of miR-21-5p.

Circulating miR-21-5p and miR-126-3p emerge as dynamic biomarkers of systemic inflammatory/angiogenic status. Their expression levels in CACs from T2DM with MACE suggest a shift from a proangiogenic to a proinflammatory profile.

## INTRODUCTION

Type 2 diabetes mellitus (T2DM) is an increasingly serious public health problem, since the number of affected adults and the proportion of T2DM patients with diabetic complications is expected to grow substantially in future decades [1]. The common and severe complications of T2DM are silent killers and are often irreversible at the time of diagnosis [2]. Genetic variability does not account for the current T2DM epidemic nor for its complications. Therefore, innovative strategies for stratifying patients with T2DM complications using blood-based biomarkers are being sought all over the world [3, 4]. The underlying pathogenesis of T2DM complications is not easily elucidated, because diabetic complications are characterized by heterogeneous phenotypes. A recent meta-analysis of randomised controlled trials has demonstrated that the benefit-risk ratio of intensive glucose lowering treatment, administered to prevent diabetic vascular complications, remains uncertain [5]. This suggests that the parameters currently used to monitor T2DM progression do not adequately predict the probability of developing complications. It is therefore of the utmost importance to identify new prognostic biomarkers capable of establishing evidence-based clinical practice recommendations for patients who are at increased risk of T2DM-related complications. Recently, epigenetic alterations have emerged as key factors in age-related diseases (ARDs), including T2DM and its complications [3, 6]. Factors associated with diabetic complications, including hyperglycaemia, oxidative stress and inflammatory factors, can modulate epigenetic mechanisms, altering gene expression in target cells, especially endothelial and immune cells. MicroRNAs (miRNAs) are a broad class of small, non-coding RNAs involved in the modulation of gene expression, either silencing or activating transcription *via* sequence-specific DNA/RNA binding [7]. Initially believed to leak passively from dead cells, miRNAs released into the bloodstream are emerging as an organized and controlled secretion process involving different shuttles [8-13]. Such mediators of epigenetic information have been extensively investigated in recent years as potential biomarkers of ARDs, including T2DM [14-19].

MiR-21 and miR-126 - now miR-21-5p and miR-126-3p, based on the latest miRBase release (V.21) - are expressed mainly by cells involved in the modulation of inflammatory response and vascular homeostasis, and therefore are expected to be significantly modulated in T2DM and its complications [20]. MiR-126-3p is involved in modulating vascular regeneration and mobilization of hematopoietic stem/progenitor cells, and was therefore classified as an “angiomiR”; it was one of the first miRNAs to be found in reduced circulating concentrations in T2DM patients [14-16]. Its downregulation has been detected in endothelial tissues of diabetic mice with peripheral artery disease as well as in endothelial progenitor cells from diabetic patients and in human umbilical vein endothelial cells (HUVECs) cultured under hyperglycaemic conditions [16, 21, 22].

MiR-21-5p was initially classified as an “oncomiR”, since it is deregulated in almost all cancer types [23]. More recently it has also been implicated in other roles, including the modulation of inflammation and tissue repair, and has therefore also been classified as an “inflammamiR” [24-30].

We focused our study on miR-126-3p and miR-21-5p levels in plasma and CACs from healthy subjects and diabetic patients, to investigate their potentiality as diabetic biomarkers in relation to the main diabetic complications.

## RESULTS

The chemical-clinical, and anthropometric characteristics of the studied groups, 107 healthy CTR subjects and 193 T2DM patients, 76 patients without (T2DM NC) and 117 patients with (T2DM C) diabetic complications, are reported in Table 1. Since T2DM group included a greater proportion of males than the CTR group, T2DM C patients were older than CTR subjects and males were a greater proportion than among T2DM NC patients as well as CTR subjects, all subsequent analyses were adjusted for age and sex.

**Table 1:** Chemical, clinical, and anthropometric characteristics of CTR subjects and patients without and with T2DM complications

Variables	CTR (N=107)	T2DM NC (n=76)	T2DM C (n=117)
Age, (yrs)	64.25±7.56	65.56±6.96	66.51±7.48#
Males, n (%)	49 (45.79)	36 (47.37)	69 (58.97)*#
BMI, kg/m2	26.67±5.4	28.47±4.34	28.57±3.46*
Glucose, mg/dL	92.23±8.41	154.63±40.78*	178.89±56.78*#
HbA1c, %	5.96±0.41	7.34±1.28*	7.77±1.19*#
Insulin, mcU/mL	5.66±3.75	6.63±4.65	6.78±4.73
HDL cholesterol, mg/dL	59.29±15.34	55.94±17.85	51.43±15.14#
Total cholesterol, mg/dL	212.72±42.21	215.17±37.35	202.19±39.20
Creatinine, mg/dL	0.82±0.22	0.84±0.17	1.01±0.43*#
Hs-CRP, mg/L	2.52±3.77	3.79±4.25	3.54±4.24
MiR-126-3p, RE	0.33±0.31	0.24±0.24*	0.23±0.19*
MiR-21, RE	0.43±0.42	0.33±0.42*	0.31±0.34*

T2DM NC: patients without diabetic complications; T2DM C: patients with diabetic complications; RE=relative expression. The chi square test was applied for dichotomous variables. ANCOVA adjusted for age and sex or for variables significantly correlating with miRNAs was applied for continuous variables: *p<0.05, reference group: CTR; #p<0.05, reference group: T2DM NC (diabetic patients without diabetic complications).

**Figure 1:**
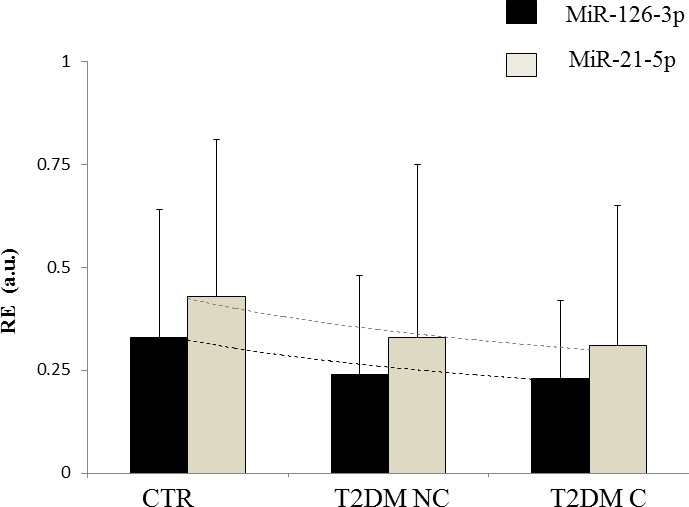
Circulating levels of miR-21-5p and miR-126-3p in CTR subjects and T2DM patients Linear trend for miR-126-3p, F = 9.51, *p* = 0.002; linear trend for miR-21-5p: F = 5.33, *p* = 0.02. CTR: healthy control subjects; T2DM NC: patients without diabetic complications; T2DM C: patients with diabetic complications. RE = relative expression. Dotted line shows the exponential trend.

MiR-126-3p and miR-21-5p declined significantly from CTR to T2DM NC and T2DM C (linear trend for miR-126-3p, F = 9.51, *p* = 0.002; linear trend for miR-21-5p: F = 5.33, *p* = 0.02; miR-126, ANCOVA adjusted for age and sex; F-test = 3.82, *p* = 0.023; miR-21-5p, ANCOVA adjusted for age, sex, glucose, and HbA1c; F-test = 4.52, *p* = 0.012) (Figure 1). However, the differences in miR-21-5p and miR-126-3p levels were not significant between patients without and with diabetes complications (ANCOVA adjusted for age, sex, glucose and HbA1c: F-test = 0.005, *p* = 0.945 and F-test = 0.612, *p* = 0.435, respectively).

Analysis in relation to the different T2DM complications showed no significant differences for neuropathy, nephropathy, chronic renal failure, retinopathy, or lower limb arteriopathy (Table 2). However, the circulating levels of both miRNAs were significantly different between patients with and without MACE (miR-21-5p: 56 patients with *vs.* 137 patients without MACE, 0.46±0.44 *vs*. 0.26±0.33, *p* < 0.001; miR-126-3p: 0.21±0.21 *vs*. 0.28±0.22, *p* = 0.032) (Table 2). Multivariate covariance analysis with miR-21-5p and miR-126-3p as the dependent variables confirmed the significance of MACE complications (Wilk's lambda = 0.943, *p* = 0.007).

**Table 2:** Circulating miR-21-5p and miR-126-3p levels in T2DM patients with different diabetic complications

	MiR-21 levels		
Diabetes complications	Patients with a specific complication *vs*. all the other patients	Mean ± SD	*p*
neuropathy	37 *vs.* 156	0.21±0.27 vs. 0.34±0.39	0.089
nephropathy	27 *vs.* 166	0.26±0.28 vs. 0.33±0.39	0.489
chronic renal failure	10 *vs.* 183	0.39±0.41 vs. 0.31±0.38	0.375
retinopathy	84 *vs.* 109	0.27±0.30 vs. 0.35±0.43	0.165
lower limb arteriopathy	11 *vs.* 182	0.37±0.33 vs. 0.31±0.38	0.534
MACE	56 *vs.* 137	0.46±0.44 vs. 0.26±0.33	**<0.001**
	**MiR-126 levels**		
neuropathy	37 *vs.* 156	0.18±0.20 vs. 0.24±0.22	0.122
nephropathy	27 *vs.* 166	0.20±0.16 vs. 0.23±0.22	0.512
chronic renal failure	10 *vs.* 183	0.23±0.19 vs. 0.23±0.22	0.993
retinopathy	84 *vs.* 109	0.23±0.20 vs. 0.23±0.23	0.863
lower limb arteriopathy	11 *vs.* 182	0.23±0.21 vs. 0.29±0.24	0.351
MACE	56 *vs.* 137	0.21±0.21 vs. 0.28±0.22	**0.032**

ANCOVA adjusted for age, sex, and number of complications. Levels lower than 0.05 in bold.

**Figure 2:**
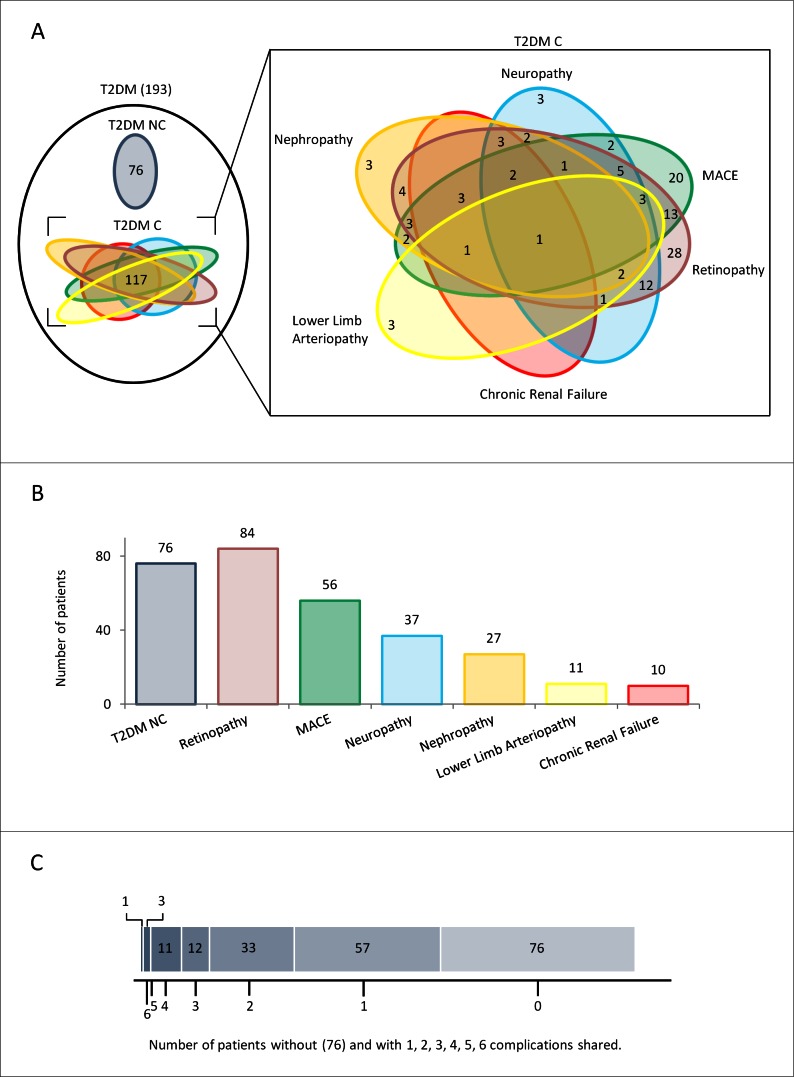
Overview of diabetic patients without or with complications **A.** Venn diagramm showing the overlaps between different complications (T2DMC). **B.** Histogram displaying the size of each group of T2DM. **C.** Charts showing number of T2DM patients without complications and with one or more complications.

**Figure 3:**
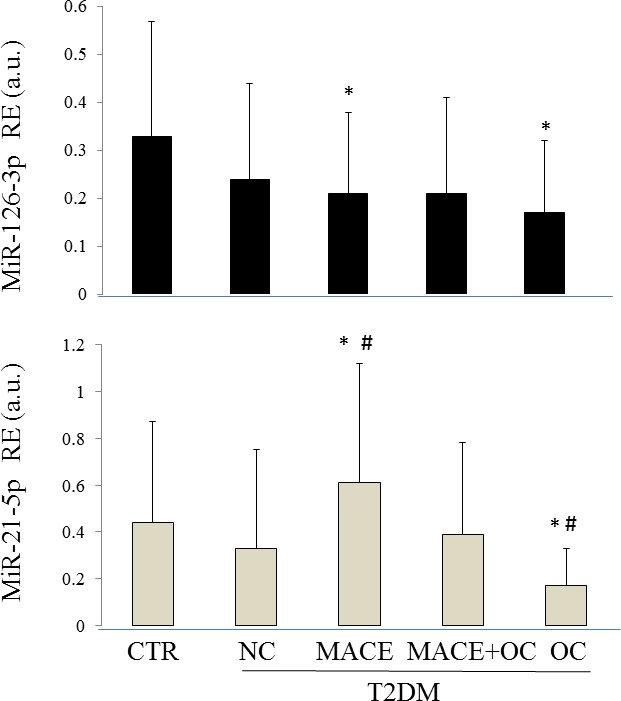
Circulating levels of miR-21-5p and miR-126-3p in CTR and T2DM patients CTR: healthy control subjects; T2DM NC: patients without diabetic complications; T2DM C: patients with diabetic complications; RE = relative expression; MACE: T2DM patients with a previous MACE; MACE+OC (other complications): T2DM patients with a previous MACE and at least another diabetic complication; OC (other complications): T2DM patients with complications other than MACE. RE: relative expression; CTR: healthy control subjects; NC: diabetic patients without diabetic complications; MACE: T2DM patients with a previous MACE; MACE+OC (other complications): T2DM patients with a previous MACE and at least another diabetic complication; OC (other complications): patients with diabetic complications other than MACE. MiR-21-5p: ANCOVA adjusted for age, sex, BMI, glucose, and HbA1c; F-test = 6.44, *p* < 0.001; ANCOVA adjusted for age and sex; F-test = 8.375, *p* < 0.001. MiR-126-3p: ANCOVA adjusted for age, sex, glucose, and HbA1c; F-test = 4.55, *p* = 0.01; miR-126-3p: ANCOVA adjusted for age and sex; F-test = 6.655, *p* = 0.002. #Bonferroni's correction, alpha = 0.05/10, *p* < 0.005, reference group: T2DM NC; *Bonferroni's correction, alpha = 0.05/10, p < 0.005, reference group: CTR.

Since among the 117 T2DM C patients enrolled for our study, 60 patients had more than one complications (Figure 2), to confirm the significant differences in circulating miR-21-5p and miR-126-3p found in patients with MACE compared with those suffering from other complications, the total 193 T2DM patients were divided into 4 groups: no complications (NC, *n* = 76), MACE only (MACE, *n* = 18); MACE and other complications (MACE+OC, *n* = 38); complications other than MACE (OC, *n* = 62). Circulating miR-21-5p and miR-126-3p levels were significantly different in the 4 groups of T2DM patients (Figure 3) (ANCOVA adjusted for age, sex, glucose, and HbA1c; miR-21-5p: F-test = 8.375, *p* < 0.001; miR-126-3p: F-test = 6.655, *p* = 0.002).

MiR-21-5p levels remained significantly higher in patients with MACE and significantly lower in diabetic patients + OC compared with patients without complications even after application of Bonferroni's correction, (*p* < 0.005).

Circulating miR-21-5p and miR-126-3p were compared also between CTR and T2DM patients groups and were found to be significantly different (ANCOVA adjusted for age, sex, BMI, glucose, and HbA1c; circulating miR-21-5p: F-test = 6.44, *p* < 0.001; circulating miR-126-3p: F-test = 4.55, *p* = 0.001). Application of Bonferroni's correction using CTR as reference group confirmed that miR-21-5p levels were significantly higher and miR-126-3p levels were significantly lower in patients with MACE. MiR-126-3p and miR-21-5p levels were both significantly lower in patients with complications other than MACE (T2DM + OC).

Correlation analysis, controlled for sex and age, between circulating miR-21-5p and miR-126-3p and the variables that were found to be significantly different between T2DM patients and healthy CTR showed weak but significant positive correlation between miR-21-5p and BMI (partial correlation coefficient, PCC; 0.12, *p* = 0.049) and a weak but significant negative correlation between miR-126-3p and glycaemia parameters (miR-126-3p/glucose, PCC, −0.13, *p* = 0.038; miR-126-3p/HbA1c, PCC, −0.13, *p* = 0.033). Notably, a positive correlation was observed between circulating miR-21-5p and glycaemia parameters (glucose and HbA1c levels) when correlation analysis was confined to T2DM patients (miR-21/glucose, PCC, 0.14, *p* = 0.049; miR-21-5p/HbA1c, PCC, 0.15, *p* = 0.045).

Since it has been reported that aspirin treatment may affect circulating levels of miRNAs [31], we asked our MACE patients whether they were taking aspirin treatment and found that they accounted for only 3 %.

**Figure 4:**
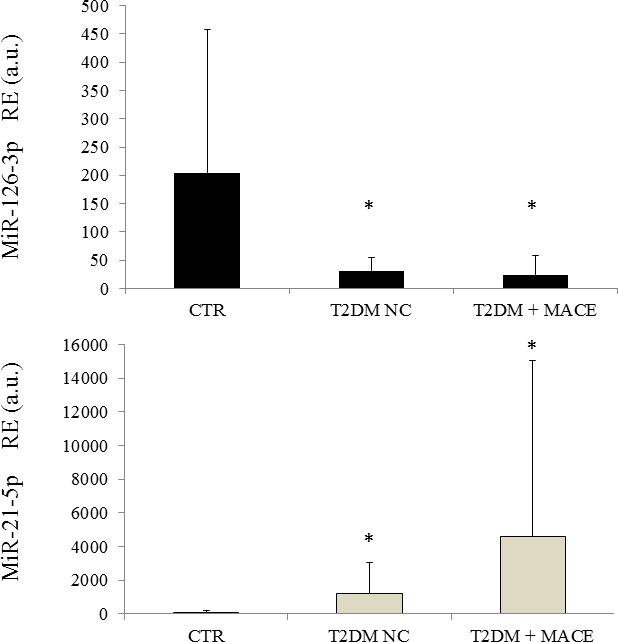
MiR-21-5p and miR-126-3p expression levels in CACs from CTR, T2DM NC and T2DM +MACE patients T2DM NC: patients without diabetic complications; T2DM + MACE: T2DM patients with a previous MACE; RE = relative expression.

To confirm the results obtained on plasma we analysed miR-126-3p and miR-21-5p expression levels in CACs from a subgroups of CTR, T2DM NC and T2DM + MACE. MiR-126-3p levels were significantly lower in CACs from T2DM NC and T2DM+MACE compared to CTR and miR-21-5p levels were higher in CACs from T2DM, both T2DM+MACE and T2DM NC, compared with CTR (Figure 4). Notably, the highest expression levels of miR-21 were observed in CACs from T2DM+MACE patients.

## DISCUSSION

Fasting blood glucose is the current diagnostic criterion for diabetes, being globally accepted for almost a decade. However, information regarding insulin resistance and type 2 diabetes has increased exponentially during the last years, suggesting the existence of complex networks that regulate energy balance, in close association with the immune system and chronic inflammation [32]. A broader view of this paradigm, prompted us to research for new potential biomarkers, such as circulating miR-126-3p and miR-21-5p, since these miRNAs were associated with endothelial dysfunction and inflammation, two milestones of T2DM progression. According with previous results, we found that both miRNAs levels decreased from CTR subjects to diabetic patients [15]. An innovative aspect of our study was to analyse circulating miRNAs levels according to the presence of diabetes complications as a whole and grouped as specific complications. We observed that miR-126-3p and miR-21-5p levels declined significantly from CTR to T2DM non complicated and T2DM complicated patients. When the analysis focused on patients with specific complications, significantly higher miR-21-5p and lower miR-126-3p levels were seen in T2DM patients with previous episode of myocardial infarction (MACE). These observations are in accordance with the results of a previous prospective study showing that higher circulating and microvesicles-contained miR-126-3p levels are protective against MACE [20]. Notably, we analysed in our retrospective study only a group of T2DM patients survived to the previous MACE.

Moreover, our and others previous data suggest that MACE is a potent inducer of miR-21-5p release in the circulation [25, 33-35]. MiR-21 transfer/hyperexpression has been hypothesized as mechanism promoting cardiomyocyte survival during acute ischemic events, and cardiac fibroblasts proliferation after the acute event [36-38].

However, it was recently highlighted an link between miR-21 expression levels and angiogenic properties of endothelial cells; miR-21 hyper-expression suppresses endothelial progenitor cells (EPC) proliferation [39] and contribute to endothelial cell senescence [40]. Moreover, we observed a significant positive correlation between circulating miR-21-5p and BMI. Recently it was reported that obesity is related to miR-21-5p expression [41] and to pro-angiogenic potential inducing a modulation of miRNAs secretion [42], suggesting a potential link between circulating miR-21 levels, BMI and pro-angiogenic properties.

When we assessed miR-126-3p and miR-21-5p levels in CACs, a reduced miR-126-5p levels and an increased miR-21-3p expression levels were observed in T2DM patients (both T2DM NC and T2DM + MACE) compared to CTR. Previous data on endothelial progenitor cells from diabetic patients shoved a reduced miR-126 expression [24], and an overexpression of miR-21 was reported in response to high glucose in endothelial cells [43].

Since miR-21 and miR-126 modulate inflammation and angiogenic pathways, our results suggest that CACs from T2DM are “proinflammatory cells” rather than “proangiogenic cells”. These conditions could depend from an increased “senescence status” of these circulating cells reached under hyperglycaemic conditions. The exposure to high glucose levels could induce the accumulation of dysfunctional endothelial senescent cells in T2DM patients shifting the transcriptional program of CACs from a “pro-angiogenic” to a “pro-inflammatory” profile, and increasing in turn the risk of developing micro and macrovascular complications. Our previous observation that telomere shortening in circulating cells from T2DM patients is related to the number of diabetic complications and that senescent CACs are present in patients with previous cardiac ischemic events [44, 45], further support this hypothesis.

## LIMITATION

Since several tissues are involved in diabetic complications, it is conceivable that all provide a relative contribution to circulating miRNA signatures. However, analysing plasma content of miRNAs it is impossible to establish which cell type contributed exactly to the circulating levels of a specific miRNA.

Further studies are required to identify and validate circulating miRNA-based signatures associated with T2DM complications in longitudinal studies.

## CONCLUSIONS

Epigenetic mechanisms, including miRNA deregulation, probably partly explain the increased risk of developing diabetic complications over time despite multifactorial interventions with glucose-lowering, lipid-lowering and anti-hypertensive drugs [46, 47]. This study provides proof of principle that circulating-based miRNAs signatures are dynamic biomarkers of the health status as a whole, and therefore significant modulations are expected depending from comorbidities.

We recommend that the presence of comorbidities must be taken into account when circulating miRNA-based signatures are proposed as diagnostic or prognostic biomarkers for the major ARDs.

## MATERIALS AND METHODS

### Patients

A total of 193 T2DM patients from central Italy and 107 healthy control subjects (CTRs) gave their informed consent to be enrolled in the study. The study protocol was approved by the Ethics Committee of INRCA-IRCCS (Ancona, Italy). T2DM was diagnosed according to American Diabetes Association Criteria [20]. Inclusion criteria were: a body mass index (BMI) < 40 kg/m^2^, age 35 to 85 years, and ability and willingness to provide a written informed consent and to comply with study requirements. The information collected included information on vital signs, anthropometric data, medical history and behaviours, and exercise.

The presence/absence of diabetic complications was established as follows:

- retinopathy was defined as dilated pupils detected on fundoscopy and / or fluorescence angiography;
- incipient nephropathy was a urinary albumin excretion rate > 30 mg / 24 h and normal creatinine clearance;
- chronic renal failure was defined as an estimated glomerular filtration rate < 60 mL / min per 1.73 m^2^;
- neuropathy was established by electromyography;
- ischemic heart disease was diagnosed by clinical history and / or ischaemic electrocardiographic alterations; these patients had had ST- or non-ST elevation myocardial infarction, which was defined as a major acute cardiac event (MACE). Mean time from the MACE was 9±8 years;
- peripheral vascular disease, including arteriosclerosis obliterans and cerebrovascular disease, was diagnosed based on history, physical examination, and Doppler imaging.

Of the 193 T2DM patients with at least one complication, 37 had neuropathy, 11 had lower limb arteriopathy, 56 had MACE, 27 had nephropathy, 10 had chronic renal failure, and 84 had retinopathy.

Control samples (CTR) were selected among the husbands and wives of T2DM patients enrolled for the study. As microRNAs are epigenetic biomarkers, their expression is expected to be related with lifestyle and environment. Therefore, the choice of CTR among people living with diabetic patients is expected to minimize the effect of environment as confounding factor.

All study subjects reported dietary habits consistent with a Mediterranean-style diet.

### Laboratory assays

Blood concentrations of total and HDL cholesterol, triglycerides, fasting glucose, HbA1c, fasting insulin, creatinine, and hs-CRP were measured by standard procedures.

### Circulating Angiogenic Cells (CACs) isolation

CACs were isolated from 15 mL of heparinized peripheral blood. Peripheral blood mononuclear cells (PBMC) were isolated by density-gradient centrifugation with Ficoll (Ficoll-Paque™ PLUS, GE Helathcare Bio-Sciences Uppsala-Sweden), within 2 hours after blood collection. 5×10^6^ PBMC were plated on 24-well fibronectin-coated plate (BD Biosciences, Mountain View, CA) and maintained in endothelial basal medium (EBM; Clonetics-Lonza, Walkersville, MD USA) supplemented with EGM SingleQuots and 20% FCS for 4 days. After 4 days in culture, non-adherent cells were removed by PBS wash, while adherent cells were lysed directly in the culture wells for RNA purification according to the Total RNA Extraction kit (NORGEN-Germany). The CAC phenotype was confirmed in fluorescence microscopy by cellular uptake of acetylated LDL (DiI-acLDL, Molecular probes) and binding of FITC-conjugated lectin from Ulex europeus (UEA-1, Sigma).

### RNA isolation and RT-qPCR assay

Prior to any therapeutic procedures, 1 ml of whole blood from each participant was collected into EDTA tubes after overnight fasting. Samples were immediately centrifuged at 3000 g for 10 min at room temperature, then centrifuged at 10,000 g for 5 min at 4°C, and subsequently stored at −80°C until analysis. Samples were frozen within 3 h of plasma separation.

After two subsequent spins, total RNA was extracted from 100 μl of plasma using an RNA purification kit (Norgen Biotek Corporation, Thorold, ON, Canada) that isolates enriched miR species. The synthetic *Caenorhabditis elegans* miR, cel-miR-39, was spiked into plasma before RNA extraction. Only samples with cel-miR-39 recovery > 95 % were used in subsequent analyses.

MiRNAs were reverse-transcribed with the TaqMan MicroRNA Reverse Transcription Kit (Life Technologies, Carlsbad, CA, United States) as recommended by the manufacturer. MiRNA-specific TaqMan MicroRNA Assays (Life Technologies) were used for plasma miRNAs.

Data were analysed by the 2^−ΔCt^ method. Relative expression corresponded to the 2^−ΔCt^ value. For analysis of miRNA expression levels, external normalization to cel-miR-39 was applied.

### Statistical analysis

Summarized data are shown as mean ± SD or as frequency (%). Analysis of covariance (ANCOVA) was used to compare the mean differences in chemical, clinical, and anthropometric variables after adjustment for age and sex (CTR subjects, and patients without and with diabetes complications). Multivariate analysis of covariance with miR-21-5p and miR-126-3p as the two dependent variables was performed to confirm the statistical significance of MACE complications.

Partial correlation, adjusted for age and sex, was used to test for correlations between miRNAs and the others variables. The association of T2DM complications with circulating miR-21-5p and miR-126-3p was also assessed for linear trend.

Data analysis was performed using IBM SPSS Statistics for Windows, version 20 (IBM Corp, Armonk, NY, USA). The significance level was a P value < 0.05. Bonferroni's correction for multiple testing was applied, alpha = 0.05/10, *p* < 0.005.
